# P-159. Easy Diagnosis of Typhoid Using CBC and LFT Tests

**DOI:** 10.1093/ofid/ofaf695.383

**Published:** 2026-01-11

**Authors:** Sherlin M S, Suresh Kumar Dorairajan, H Hemanth

**Affiliations:** Sri venkateswara college of pharmacy, Chittoor, Andhra Pradesh, India; Apollo hospitals,Vanagaram, Chennai, Tamil Nadu, India; Sri venkateswara college of pharmacy, Chittoor, Andhra Pradesh, India

## Abstract

**Background:**

Typhoid fever is still a major health issue in many parts of the world. Traditional tests like blood culture and the Widal test are often slow or unreliable. This study explores whether Complete Blood Count (CBC) and Liver Function Tests (LFTs) can help in the early and affordable diagnosis of typhoid.

**Figure:**
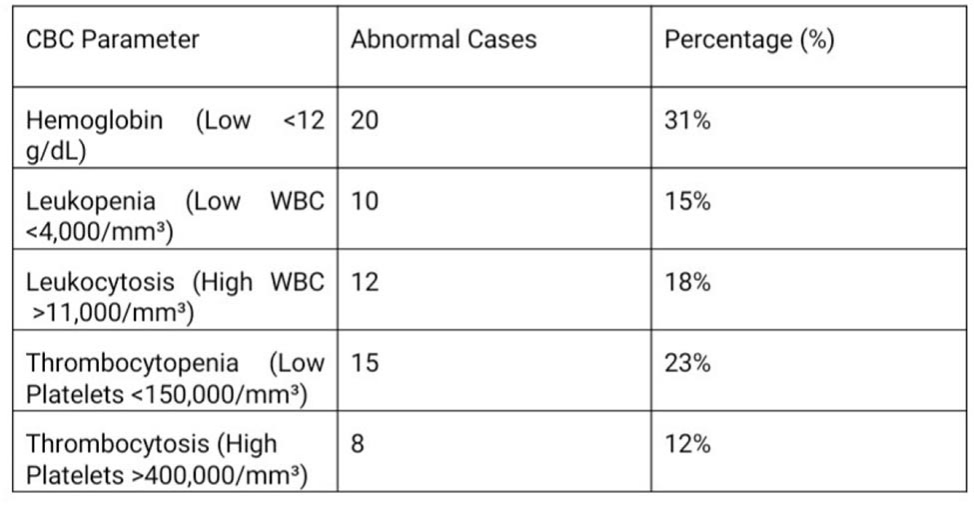
CBC Parameters Noted

**Figure:**
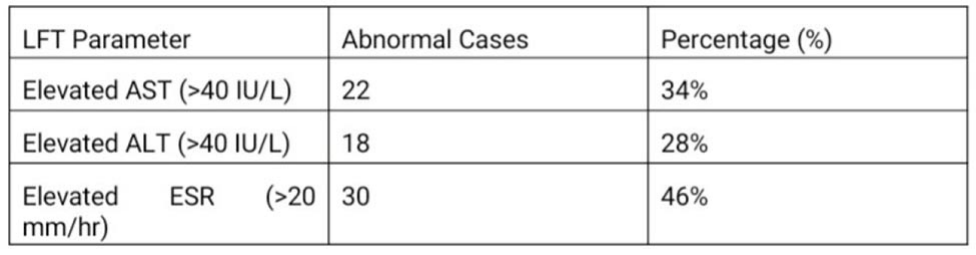
LFT Parameters observed

**Methods:**

A retrospective review was done on patients with symptoms like fever, weakness, and abdominal pain. CBC and LFT results were collected and compared with confirmed typhoid cases. Key parameters included hemoglobin, WBC and platelet counts, ESR, and liver enzymes (ALT, AST, bilirubin).

**Results:**

Out of 90 confirmed typhoid patients (ages 1–82), many had abnormal lab results. Low hemoglobin was seen in 31%, low WBC in 15%, high WBC in 18%, and low platelets in 23% [Table 1]. Elevated AST and ALT were found in 34% and 28% of cases, respectively, and 46% had high ESR [Table 2]. A few severe cases had complications, including two deaths.

**Conclusion:**

CBC and LFT tests provide valuable early diagnostic clues for typhoid fever. A combination of leukopenia, thrombocytopenia, high ESR, and elevated liver enzymes can serve as a rapid, accessible, and cost-effective preliminary diagnostic approach before confirmatory testing. This can aid in early intervention and better clinical management, especially in resource-limited settings.

**Disclosures:**

All Authors: No reported disclosures

